# Clinical and functional aspects of body balance in elderly subjects with benign paroxysmal positional vertigo

**DOI:** 10.5935/1808-8694.20130027

**Published:** 2015-11-02

**Authors:** Daniela Patricia Vaz, Juliana Maria Gazzola, Solange Martiliano Lança, Ricardo Schaffeln Dorigueto, Cristiane Akemi Kasse

**Affiliations:** a MSc in Body Balance Rehabilitaton and Social Inclusion at UNIBAN (Bandeirantes University of São Paulo) (Coordinator of the Imaging graduate program at FAMESP - Método College of São Paulo. Professor in the undergraduate and graduate programs at FAMESP); b PhD in Sciences at the Department of Otorhinolaryngology and Head and Neck Surgery - UNIFESP (Physical Therapist, Professor in the Body Balance Rehabilitaton and Social Inclusion graduate program at UNIBAN - Bandeirantes University of São Paulo); c MSc in Body Balance Rehabilitaton and Social Inclusion at UNIBAN (Speech and Hearing Therapist); d PhD in Sciences at the Department of Otorhinolaryngology and Head and Neck Surgery - UNIFESP (Professor in the Body Balance Rehabilitaton and Social Inclusion graduate program at UNIBAN - Bandeirantes University of São Paulo)

**Keywords:** aged, dizziness, patient positioning

## Abstract

Benign paroxysmal positional vertigo (BPPV) may compromise the balance of elderly subjects.

**Objective:**

To observe the effects of the Epley maneuver in elderly subjects with BPPV and assess clinical and functional aspects of body balance.

**Method:**

This is a prospective clinical study. Patients diagnosed with BPPV (Dix-Hallpike test) were submitted to the Timed Up & Go (TUG) test, the Clinical Test of Sensory Interaction and Balance (CTSIB), and lower limb testing before and after they were repositioned using the modified Epley maneuver.

**Results:**

most subjects were females, and the group's mean age was 70.10 years (SD = 7.00). All patients had canalithiasis of the posterior canal. The following symptoms improved after the maneuver: postural instability (*p* = 0.006), nausea and vomiting (*p* = 0.021), and tinnitus (*p* = 0.003). Subjects improved their times significantly in the TUG and lower limb tests after the Epley maneuver (*p* < 0.001). Patients performed better on the CTSIB after the Epley maneuver on condition 2 (*p* < 0.003), condition 3 (*p* < 0.001), condition 4 (*p* < 0.001), condition 5 (*p* < 0.001), and condition 6 (*p* < 0.001).

**Conclusion:**

Clinical and functional aspects of body balance in elderly with BPPV improved after treatment with the modified Epley maneuver.

## INTRODUCTION

Dizziness is a highly prevalent symptom among the elderly, and has been considered as a geriatric syndrome^1^. Along with body balance alterations, they account for 5% to 10% of the visits to medical clinics every year and involve 40% of the subjects aged 40 and above^2^.

Benign paroxysmal positional vertigo (BPPV) is the most common cause of vertigo. BPPV is observed in 64/100,000 people, with even higher prevalence rates among the elderly. It is estimated that 25% of the subjects affected by dizziness aged 70 and above have BPPV. Most tend to live with it for over a year before seeking help^3^. The most widely accepted pathophysiological substrate is the one originated by the detachment of statoconia from the utricular macula that may adhere to the cupula (cupulolithiasis) or circulate freely in the endolymph in the ducts of the semicircular canals (canalithiasis)^4^, ^5^.

BPPV is characterized by intense objective or subjective rotational dizziness, usually lasting for a few seconds in sporadic spells triggered by movements of the head, in particular when hyperextending the neck, tilting the trunk forward, getting up and lying down in bed, or changing positions in bed. Patients tend to refrain from performing these movements so as to avoid episodes of vertigo, but may develop postural disor-ders^6^, body balance and functional capacity impairments and, consequently, see reductions in their quality of life. Vestibular dizziness restricts postural control, thus harming stability and body alignment and leading to balance impairment and increased risk of falls^7^. Vertigo episodes are usually accompanied by nausea, vomiting, headache, imbalance, and falls.

The Epley repositioning maneuver^8^, proposed and described in 1992 and modified by Herdman et al.^9^ in 1993, is considered by many to be the most effective maneuver used to treat BPPV. It has been indicated for patients with BPPV and canalithiasis of the posterior or anterior canal, with a reported rate of effectiveness of 70% to 100%^10^, ^11^.

Many studies have been carried out to analyze the modified Epley maneuver and its effectiveness in improving patient symptoms, quality of life, and postural control, but none tried to correlate the modified Epley maneuver to functional tests that mimic the situations experienced by the elderly in their daily lives^7^, ^12^.

The scarcity of studies on the assessment of body balance through functional tests before and after the treatment of BPPV using the modified Epley maneuver motivated the writing of this paper. This study aimed to assess the clinical and functional characteristics of body balance in elderly individuals with BPPV before and after the application of the modified Epley maneuver.

## METHOD

This longitudinal analytic descriptive study was approved by the institution's Research Ethics Committee and granted permit 104/10. The sample included patients with BPPV of both genders, aged 60 and above. The subjects enrolled in the study signed an informed consent term. The exclusion criteria comprehended the following: subjects with physical and sensory limitations such as unresponsiveness to simple verbal commands and inability to imitate movements that prevented them from performing balance tests; individuals with incapacitating diminished auditory or visual acuity even when fitted with lenses or hearing aids; patients with movement limitations or balance alterations while in an orthostatic position due to amputations of the lower limbs above the metatarsophalangeal joints; subjects with altered postural balance due to the use of lower or upper limb prosthetics; individuals unable to walk independently; subjects in wheelchairs.

Subjects submitted to body balance rehabilitation up to six months prior to the beginning of the study, individuals who took vertigo medication during the study, and patients with neck disturbances that prevented them from performing the maneuver were also excluded. Data collection was carried out between July of 2010 and July of 2011. The preliminary sample had 42 subjects, but after the application of exclusion criteria and a few drop-outs, the final sample had 30 patients. Subject ages ranged from 60 to 91 years (mean = 70.10 years; standard deviation = 7.00 years; median = 69 years). Twenty-eight (93.3%) patients were females.

**Figure 1:**
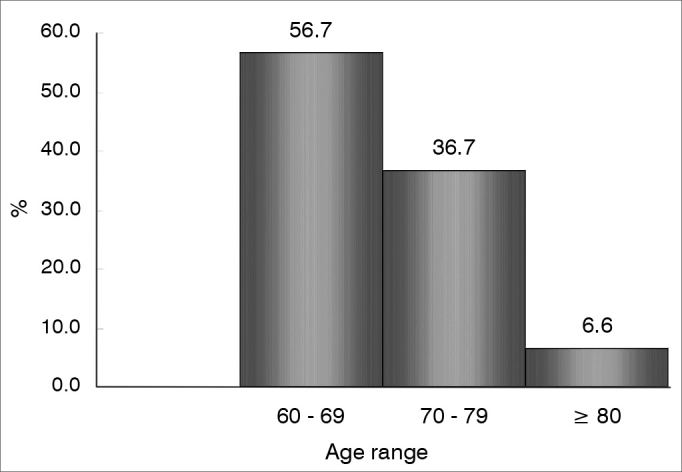
Distribution of 30 patients according to age range.

Figure 1 shows the patient age range distribution.

Subjects were assessed by ENTs from the clinic, and the diagnosis of BPPV was based on the Dix & Hallpike^13^ and the Roll^14^ tests. The collected otoneurological data included clinical and topographic diagnosis of vestibular disorder, affected canal, pathophysiological substrate, recurrence, dizziness triggering factors, time since the onset of dizzy spells, type of dizziness, duration of dizzy spells, periodicity of dizzy spells, associated symptoms, whether the subject did any physical activity, and number of maneuvers needed for symptoms and nystagmus to disappear.

One week after the medical examination, the patients were assessed for the functional aspects of body balance through the Timed Up and Go (TUG) test^15^, the Clinical Test of Sensory Interaction and Balance (CTSIB)^16^, and the lower limb test^17^.

The TUG test assesses how fast subjects can get up from a chair, walk away from the chair for three meters, walk back to the chair, and sit down. Speed is analyzed considering mobility and body balance displayed by the individuals as they perform the task. The speed with which the patients perform the test is correlated to their risk of falls. Times greater than 13.5 seconds characterize the highest risk of falls. This test was designed and adapted from the Get-up and go test^18^.

A chair, red adhesive tape to mark the course on the floor, and a stopwatch were used in this test. The rater explained and showed to the patients what the test entailed before asking them to perform the task. The test was initiated as the rater told the patients to start through a verbal command and a visual cue (lifting the arm).

The CTSIB is used to systematically test the impact of visual, somatosensory, and vestibular stimuli upon an individual's capacity of maintaining static balance, ultimately to improve the patient's re-adaptation and learning processes. The test is applied with the patient in a static position, and includes visual inputs and changes to the support surface to manipulate the subject's sensory responses and provide insight into the impact of sensory action upon postural control.

In the original test, patients were asked to stand on a surface picked by the rater. The surface area was reduced so that the patient had to stand with feet together (Romberg position). This position of the feet increases the sensitivity of balance analysis and correlates better with the support surface used in the Sensory Organization Test (SOT)^19^.

In order to avoid impact from learning upon test results, the six conditions of the CTSIB were applied randomly to the subjects and they were given one try at each condition. The conditions on a firm surface were numbered as follows: condition 1 (eyes open), condition 2 (eyes closed), and condition 3 (visual conflict dome occluding eyesight). Conditions on a compliant surface (foam) were named as follows: condition 4 (eyes open), condition 5 (eyes closed), and condition 6 (visual conflict dome). The density of the foam used in the sensory interaction test was 33 mm. The visual conflict dome measured 40 cm in diameter and 40 cm in height. Patients were asked to put it on their heads to occlude eyesight and provoke visual conflict. The dome is a static device without any special type of luminosity. Normal test results are assigned to patients able to stand for 30 seconds on each condition.

The lower limb test was designed to verify whether patients possess the muscle strength required to perform activities of daily living such as getting up from a chair or sitting down on a toilet^17^, ^20^. Lower scores may correlate to falls in elderly subjects. In the lower limb test subjects are first asked to cross their arms on their chests and sit up and down five times from a chair. Patients able to perform the task in 11.19 seconds or less are not at risk for falls; times between 11.20 and 13.69 seconds correlate with mild reduction of lower limb strength; times ranging from 13.70 to 16.69 seconds indicate moderate involvement; and times above 16.70 seconds are associated with risk of falls^21^.

The modified Epley maneuver was chosen as the treatment option for patients with vertical canal BPPV (postural restriction and bone conduction vibrator were not used). Patients had weekly treatment sessions until vertigo and positioning nystagmus disappeared^14^. When the symptoms subsided, subjects were referred to medical examination and scheduled for functional assessment the following week, to undergo the same functional tests performed before the treatment. Before and after treatment evaluations were carried out by different health care workers who were unaware of the previous test results.

All variables were initially submitted to descriptive statistical analysis. Quantitative variables were observed for their minimum, maximum, and mean values, standard deviations (SD), and medians. Absolute and relative frequencies were calculated for qualitative variables.

The Wilcoxon signed-rank test was used to compare the before and after treatment periods, as the assumed normality of the data was rejected. The McNemar test was used to test changes in the ratios. A level of significance of 5% was adopted in all tests. The following variables were statistically compared:

• Presence/absence of symptoms: Chi-square test or Fisher's exact test were used to analyze the association between frequencies of a sample with two categories, before and after treatment.
• TUG test times (before and after treatment): Wilcoxon signed-rank test.
• Time spent on the six conditions of the CTSIB (before and after treatment): Wilcoxon signed-rank test.
• Time spent on lower limb tests (before and after treatment): Wilcoxon signed-rank test.

The two stages at which patients were analyzed were called assessment and reassessment.

## RESULTS

In regards to clinical and topographic diagnosis, 30 (100%) patients had canalithiasis of the posterior semicircular canal as a pathophysiological substrate.

Considering dizziness, 17 (52.0%) patients had had dizzy spells for over five years, two (6.0%) for three to four years, seven (21.0%) for three to six months, and four (12.0%) for one to two years;

In terms of clinical complaints of BPPV, 27 (83.33%) subjects had rotational dizziness and three (16.66%) had both types of dizziness (rotational and non-rotational). Seventeen (38.88%) patients had subjective rotational dizziness, six (22.22%) had objective dizziness, and seven (38.88%) had both kinds (subjective and objective).

When duration of dizzy spells was considered, 22 (55.55%) subjects reported spells lasting seconds, five (27.77%) complained of daily durations, two (11.1%) said they lasted minutes, and one patient (5.55%) had spells that lasted for hours.

In regards to periodicity of spells, 21 (70.00%) had sporadic dizzy spells, eight (26.66%) had daily spells, and one (3.33%) had monthly episodes.

Fourteen (46.66%) patients reported that getting up or lying down in bed triggered dizzy spells; 11 (36.66%) had spells triggered when they turned their heads; nine (30.00%) when turning either while seated or standing; 10 (33.33%) when standing up from a seated position; nine (30,00%) when walking; 12 (40.00%) when anxious; 12 (40.00%) when their heads were held still in a specific position; two (6.66%) while seated or standing; 13 (43.33%) when changing positions in bed; eight (26.66%) when performing physical exercises; and 11 (36.66%) when lying in bed on one specific side of the body.

Patients improved from symptoms after treatment with a mean of 2.17 modified Epley maneuvers. The number of maneuvers needed to eliminate symptoms ranged from one to six (Table 1).

**Table 1:** Number of modified Epley maneuvers needed to cease symptoms and positioning nystagmus.

Total number of patients	Mean number of maneuvers	SD	Median	Minimum	Maximum
30	2.17	1.13	2.00	1.00	6.00

Significant differences were observed in otoneurological symptoms before and after treatment with the modified Epley maneuver, as follows: postural instability (*p* = 0.006), nausea and vomiting (*p* = 0.021), and tinnitus (*p* = 0.003) (Table 2).

**Table 2:** Presence of otoneurological symptoms associated with BPPV in 30 patients before and after treatment with the modified Epley maneuver.

	Stage				
	Assessment		Reassessment		
Symptoms	n	%	n	%	p*
Headache	17	60.7	13	46.4	0.344
Sensation of fainting	15	51.7	8	27.6	0.092
Body instability	25	86.2	15	51.7	0.006
Anxiety	21	72.4	16	55.2	0.063
Nausea/vomiting	16	55.2	8	27.6	0.021
Blacking out	14	48.3	8	27.6	0.146
Disordered sleep	20	69.0	15	51.7	0.18
Tinnitus	18	62.1	7	24.1	0.003
Hearing loss	9	31.0	7	24.1	0.727
Sensation of pressure	11	37.9	6	20.7	0.267
Memory disorders	15	51.7	13	44.8	0.687
Hypersensitivity to sound	19	65.5	14	48.3	0.302
Sweating/pallor/tachycardia	8	27.6	11	37.9	0.549
Oscillopsia	8	27.6	6	20.7	0.754

^*^ McNemar test's descriptive probability level.

The time needed to perform the TUG test and the scores of the lower limb test were significantly decreased after treatment with the modified Epley maneuver (Table 3).

**Table 3:** Descriptive values for TUG and lower limb tests during patient assessment and reassessment.

Variable	Stage	n	Mean	SD	Median	Minimum	Maximum	p*
TUG test (seconds)	Assessment	30	14.15	2.86	14.30	9.67	19.63	< 0.001
	Reassessment	3030	9.85	2.26	9.22	5.48	15.05	
Lower limb test (seconds)	Assessment		19.63	5.65	20.00	8.85	35.25	< 0.001
	Reassessment	30	13.61	3.59	13.08	7.22	19.98	

^*^ Paired *Student's t-test* descriptive probability level.

Significant differences were seen after treatment with the modified Epley maneuver in the CTSIB condition 2 (*p* = 0.003), condition 3 (*p* = < 0.001), condition 4 (*p* = < 0.001), condition 5 (*p* = < 0.001), and condition 6 (*p* = 0.001). Only condition 1 did not yield a statistically significant difference (*p* = 0.080) (Table 4).

**Table 4:** Descriptive values of the six conditions of the Clinical Test of Sensory Interaction and Balance (CTSIB) during assessment and reassessment of elderly subjects with BPPV before and after treatment with the modified Epley maneuver.

Condition	Stage	n	Mean (seconds)	SD	Median	Minimum	Maximum	p*
1	Assessment	30	29.27	2.28	30.00	19.20	30.22	0.080
	Reassessment	30	30.00	0.00	30.00	30.00	30.00	
2	Assessment	30	25.71	7.13	30.00	6.67	30.00	0.003
	Reassessment	30	30.00	0.00	30.00	30.00	30.00	
3	Assessment	30	23.70	8.31	28.03	0.00	30.00	< 0.001
	Reassessment	30	30.00	0.00	30.00	30.00	30.00	
4	Assessment	30	22.50	9.17	26.38	0.00	30.00	< 0.001
	Reassessment	30	30.00	0.00	30.00	30.00	30.00	
5	Assessment	30	20.06	10.31	22.50	0.00	30.00	< 0.001
	Reassessment	30	29.55	2.45	30.00	16.58	30.00	
6	Assessment	30	17.91	11.13	19.50	0.00	30.00	< 0.001
	Reassessment	30	29.66	1.85	30.00	20.06	30.00	

^*^ Wilcoxon signed-rank test's descriptive probability level

None of the patients had trouble in the CTSIB condition 1 (standing in an orthostatic position on a firm surface with eyes open). Subjects had some difficulty performing condition 2 (eyes closed on a firm surface), when somatosensory and proprioceptive systems were prioritized. Before treatment with the modified Epley maneuver the test was terminated with a mean of 25.71 seconds. After treatment with the maneuver, the termination time went up to a mean of 30 seconds (*p* = 0.003).

In condition 3, patients were positioned on a firm surface with a visual conflict dome on to prioritize the vestibular and proprioceptive systems while maintaining balance. The test was terminated after a mean of 23.70 seconds. After treatment with the modified Epley maneuver the mean time to termination went up to 30 seconds (*p* < 0.001).

In condition 4, subjects were assessed with eyes open standing on a compliant surface. Their somatosensory accuracy was removed and they depended on the integrity of their vestibular and visual systems to maintain balance. Before treatment with the modified Epley maneuver the test was terminated after a mean of 22.50 seconds. After treatment, the mean time to termination went up to 30 seconds (*p* <0.001).

In condition 5, individuals were tested with eyes closed standing on a compliant surface, so they could rely exclusively on their vestibular systems. After treatment with the maneuver the mean time to test termination was 30 seconds (*p* < 0.001).

In condition 6, visual and somatosensory conflicts were introduced, as patients were asked to put on the dome and to stand on a compliant surface. Mean time to test interruption was 18.15 seconds before treatment and 29.45 seconds after treatment with the maneuver (*p* < 0.001).

## DISCUSSION

The mean age of the enrolled subjects was 70.10 years. According to Parnes et al.^22^, BPPV is more prevalent in individuals aged 60 and above, who also present other comorbidities. More female subjects were included in this study, as also reported in the literature, possibly because of hormone-related factors^23^.

Cases of canalithiasis of the posterior semicircular canal prevailed, as also found by other authors^3^. Ganança et al.^24^ reported prevalence of this patho-physiological substrate and mentioned that statoconia accumulate preferably in this location by action of the gravitational force and the position of the canalithiasis in the posterior semicircular canal, as also commented by Bhattacharyya et al.^14^. The left labyrinth was more prevalently involved, with a mean of 1.33. Only one patient had both canals involved. Von Breven et al.^25^ described more cases of right labyrinth involvement (1.44 times more frequent than left side involvement), explained by the side on which patients slept. In this study, patients were not asked about the side of the bed they slept on.

The use of the modified Epley maneuver has been described in the literature as an effective and easy-to-use repositioning method, particularly in patients with posterior canal BPPV^14^, ^26^. All subjects had posterior canal BPPV and improved from symptoms after two or three treatments with the modified Epley maneuver, as similarly reported by Dorigueto et al. and Cohen^3^, ^27^. The individuals submitted to treatment with the modified Epley maneuver felt mild discomfort from the induction of symptoms. None of the subjects had cervical spine disorders, and the maneuver could be performed without motion restrictions or special care.

Dizziness, imbalance, postural instability, and falls are the most common complaints of elderly individuals with BPPV. Tinnitus, hypersensitivity to sounds, memory involvement, and disordered sleep may accompany these manifestations^28^. In this study, nineteen patients complained of tinnitus, 21 of disordered sleep, 14 of memory disorders, and 20 of hypersensitivity to sounds. According to Costa et al.^29^, the most common complaints of patients with BPPV include balance disorders lasting for hours or days, sensation of dizziness or vertigo while tilting the trunk forward, looking up, or changing positions when lying down.

After treatment with the modified Epley maneuver, the subjects improved from the following symptoms: postural instability (*p* = 0.006), nausea and vomiting (*p* = 0.021), and tinnitus (*p* = 0.003). These symptoms are directly related to BPPV, but the improvement from tinnitus was unexpected. Headache, sensation of fainting, anxiety, blacking out, disordered sleep, hearing loss, sensation of pressure in the ear, memory disorders, hypersensitivity to sounds, sweating, and oscillopsia may be related to other vestibular diseases involving the cochlea and other organs, and are not affected by the treatment.

Patients with BPPV have body imbalance^30^, ^31^, as confirmed by the results from the TUG test. After the treatment with the modified Epley maneuver, 12 of these patients had times under 11 seconds, indicating improved dynamic balance.

The lower limb test assesses muscle strength and body balance^17^. BPPV leaves patients unstable and negatively affects performance at the lower limb test. After the maneuver, patients improved their body balance and performed better at the test.

Test results have shown that body balance was impaired when patients were assessed in situations in which their visual and somatosensory functions were inaccurate or absent. The main consequence was body oscillation and unstable balance. Ricci et al.^32^ described that instability is the main problem to affect the elderly. After treatment with the modified Epley maneuver, subjects improved from symptoms.

Sensory interaction^33^ test results have shown that elderly individuals with BPPV experience impairment of the static postural control function. Symptoms subside after treatment with the modified Epley maneuver, and postural balance control is improved in conditions of somatosensory and visual conflict and visual-vestibular interaction. The authors of this study reviewed publications on static and dynamic posturography, as there are no studies on the body balance of subjects with BPPV that used the tests described herein, and found general agreement in relation to the effectiveness of the modified Epley maneuver^34^.

Posturography studies infer alterations in the dynamic^35^ and static^34^, ^36^ balance of BPPV patients based on alterations in the vestibulo-ocular reflex (VOR) and in the vestibulospinal reflex (VSR), particularly by introducing conflicts on the vestibular, somatosensory, and visual systems and producing unpleasant symptoms independent from head movement that may limit the performance of activities of daily living^37^.

The same alterations were observed in the dynamic and static balance tests carried out in this study, confirming that BPPV alters postural control of untreated patients and increases the risk of falls, body instability, and limitation of activities of daily living.

Significant functional improvement was seen after treatment with the modified Epley maneuver, as supported by the body balance test results. Epley maneuvers to reposition statoconia aim at eliminating symptoms, reducing body instability and risk of falls, and enabling patients to go back to performing activities of daily living as soon as possible^38^, ^39^.

The CTSIB, TUG, and lower limb tests can be used to easily and practically assess the static and dynamic balance of elderly subjects with BPPV. These tests can be performed using affordable materials and without high-technology equipment. Patients with unimproved test results should be suspected for other vestibular diseases or body instability arising from lack of proper treatment.

## CONCLUSION

This study showed that elderly patients with BPPV experience functional impairments related to body balance. The Epley modified maneuver can effectively resolve clinical symptoms and re-establish the functional aspects of body balance.
